# Prognosis and cure of long‐term cancer survivors: A population‐based estimation

**DOI:** 10.1002/cam4.2276

**Published:** 2019-06-17

**Authors:** Luigino Dal Maso, Chiara Panato, Stefano Guzzinati, Diego Serraino, Silvia Francisci, Laura Botta, Riccardo Capocaccia, Andrea Tavilla, Anna Gigli, Emanuele Crocetti, Massimo Rugge, Giovanna Tagliabue, Rosa Angela Filiberti, Giuliano Carrozzi, Maria Michiara, Stefano Ferretti, Rosaria Cesaraccio, Rosario Tumino, Fabio Falcini, Fabrizio Stracci, Antonietta Torrisi, Guido Mazzoleni, Mario Fusco, Stefano Rosso, Francesco Tisano, Anna Clara Fanetti, Giovanna Maria Sini, Carlotta Buzzoni, Roberta De Angelis, AIRTUM Working group, Saverio Virdone, Gemma Gatta, Manuel Zorzi, Sandra Mallone, Federica Toffolutti, Antonio Giampiero Russo, Anna Luisa Caiazzo, Lucia Mangone, Walter Mazzucco, Fabio Pannozzo, Paolo Ricci, Gemma Gola, Giuseppa Candela, Antonella Sutera Sardo

**Affiliations:** ^1^ Cancer Epidemiology Unit Centro di Riferimento Oncologico di Aviano (CRO) IRCCS Aviano Italy; ^2^ Veneto Tumour Registry Azienda Zero Padua Italy; ^3^ National Center for Prevention and Health Promotion Italian National Institute of Health (ISS) Rome Italy; ^4^ Evaluative Epidemiology Unit, Department of Preventive and Predictive Medicine Fondazione IRCCS Istituto Nazionale dei Tumori Milan Italy; ^5^ Institute for Research on Population and Social Policies National Research Council Rome Italy; ^6^ Romagna Cancer Registry Istituto Scientifico Romagnolo per lo Studio e la Cura dei Tumori (IRST), IRCCS and Azienda Usl della Romagna Meldola (Forlì) Italy; ^7^ Department of Medicine (DIMED) University of Padua Padua Italy; ^8^ Lombardy Cancer Registry-Varese Province, Cancer Registry Unit, Department of Research Fondazione IRCCS Istituto Nazionale Tumori Milan Italy; ^9^ Liguria Cancer Registry, Clinical Epidemiology IRCCS Policlinico San Martino Genova Italy; ^10^ Modena Cancer Registry, Public Health Department AUSL Modena Modena Italy; ^11^ Parma Cancer Registry, Oncology Unit Azienda Ospedaliera Universitaria di Parma Parma Italy; ^12^ Romagna Cancer Registry ‐ Section of Ferrara. Local Health Unit University of Ferrara Ferrara Italy; ^13^ North Sardinia Cancer Registry Azienda Regionale per la Tutela della Salute Sassari Italy; ^14^ Cancer Registry ASP Ragusa Victoria Italy; ^15^ Public Health Section, Department of Experimental Medicine University of Perugia Perugia Italy; ^16^ Registro Tumori Integrato Catania‐Messina‐Siracusa‐Enna Catania Italy; ^17^ South‐Tyrol Tumor Registry Bolzano Italy; ^18^ Cancer Registry of ASL Napoli 3 Sud Napoli Italy; ^19^ Registro Tumori Piemonte Provincia di Biella CPO Biella Italy; ^20^ Cancer Registry of the Province of Siracusa Local Health Unit of Siracusa Siracusa Italy; ^21^ Sondrio Cancer Registry, Epidemiology unit ATS della Montagna Sondrio Italy; ^22^ Nuoro Cancer Registry ASSL Nuoro/ATS Sardegna Nuoro Italy; ^23^ Tuscany Cancer Registry Clinical and Descriptive Epidemiology Unit, Cancer Prevention and Research Institute (ISPO) Florence Italy; ^24^ AIRTUM Database Florence Italy; ^25^ Department of Oncology and Molecular Medicine Italian National Institute of Health (ISS) Rome Italy

**Keywords:** cancer cure, Italy, population‐based cancer registries, prevalence, survival

## Abstract

**Background:**

Increasing evidence of cure for some neoplasms has emerged in recent years. The study aimed to estimate population‐based indicators of cancer cure.

**Methods:**

Information on more than half a million cancer patients aged 15‐74 years collected by population‐based Italian cancer registries and mixture cure models were used to estimate the life expectancy of fatal tumors (LEFT), proportions of patients with similar death rates of the general population (cure fraction), and time to reach 5‐year conditional relative survival (CRS) >90% or 95% (time to cure).

**Results:**

Between 1990 and 2000, the median LEFT increased >1 year for breast (from 8.1 to 9.4 years) and prostate cancers (from 5.2 to 7.4 years). Median LEFT in 1990 was >5 years for testicular cancers (5.8) and Hodgkin lymphoma (6.3) below 45 years of age. In both sexes, it was ≤0.5 years for pancreatic cancers and NHL in 1990 and in 2000. The cure fraction showed a 10% increase between 1990 and 2000. It was 95% for thyroid cancer in women, 94% for testis, 75% for prostate, 67% for breast cancers, and <20% for liver, lung, and pancreatic cancers. Time to 5‐year CRS >95% was <10 years for testis, thyroid, colon cancers, and melanoma. For breast and prostate cancers, the 5‐year CRS >90% was reached in <10 years but a small excess remained for >15 years.

**Conclusions:**

The study findings confirmed that several cancer types are curable. Became aware of the possibility of cancer cure has relevant clinical and social impacts.

## 1 INTRODUCTION

The number of people living after a cancer diagnosis showed an approximately 3% annual increase in the USA, Italy, and UK.^1^, ^2^, ^3^ This trend is mainly powered by the increasing number of new diagnoses because of population aging, and improved survival associated with advanced treatments and early diagnosis. Patients living after a cancer diagnosis (ie, prevalent cases) include those currently in treatment; those who have become cancer‐free but still have a measurable excess risk of recurrence or death; and patients who can be considered “cured”, as they have reached the same death rates of the general population.^4^ Notably, nearly four million people are expected to be living after a cancer diagnosis in Italy in 2020 (ie, one out of 17 Italians).^1^ In addition, patients who were diagnosed since ≥15 years represented one fifth (20%) of all Italian prevalent cases in 2010, and this proportion is projected to reach approximately 40% in 2020.^1^ Notwithstanding, relatively few studies have attempted to categorize prevalent cancer patients according to the probability of being cured.^4^, ^5^, ^6^, ^7^, ^8^, ^9^, ^10^

The aim of the present study was to provide reliable and updated estimates for Italian patients of three indicators that are still lacking in current cancer statistics,^11^, ^12^ that is, long‐term survival and cure, according to cancer type, sex, and age. These indicators are meant to provide helpful information to public health operators in treatment evaluation, to oncologists in planning patients’ follow‐up,^13^, ^14^, ^15^ to policy makers for an evidence‐based planning of financial resources allocation, and, most of all, they could be of special interest to the increasing number of people living after a cancer diagnosis.^1^, ^16^

## 2 MATERIALS AND METHODS

This study used data collected by 8 population‐based Italian cancer registries,^11^ which agreed to participate in the study, with at least 18 years of cancer registration as of 31 December 2011 (ie, Ferrara, Genova, Modena, Parma, Ragusa, Sassari, Varese, and Veneto, representing 10% of the entire Italian population in 2010).^11^, ^17^

The study included all malignant tumors (ICD‐10: C00‐C43, C45‐C96), and those with benign/uncertain behavior or in situ bladder cancers. Nonmelanoma skin cancers (ICD‐10 C44) were excluded. ICD‐O‐3 classification was used to identify subtypes (Table 1).

**Table 1:** Cancer cases (N) and crude incidence rates (CIR), Italian cancer registries areas 1985‐2011

Cancer type	ICD10/ICDO3	BOTH SEXES	MEN						WOMEN					
			Age 15‐74 years		15‐44	45‐54	55‐64	65‐74	Age 15‐74 years		15‐44	45‐54	55‐64	65‐74
		N	N	CIR	N	N	N	N	N	CIR	N	N	N	N
Oral cavity and pharynx	C01‐14	12,536	9,663	19.7	760	2,139	3,599	3,165	2,873	5.7	374	562	889	1,048
Esophagus	C15	4,678	3,922	8.0	131	661	1,425	1,705	756	1.5	27	104	243	382
Stomach	C16	19,407	12,701	25.9	551	1,438	3,895	6,817	6,706	13.2	437	843	1,795	3,631
Colon	C18	41,089	23,228	47.3	860	2,565	7,403	12,400	17,861	35.2	838	2,489	5,530	9,004
Rectum	C19‐20	17,782	10,995	22.4	418	1,417	3,695	5,465	6,787	13.4	328	1,068	2,160	3,231
Liver	C22	16,140	12,195	24.8	316	1,287	3,967	6,625	3,945	7.8	107	286	948	2,604
Gallbladder	C23‐24	5,119	2,286	4.7	47	233	679	1,327	2,833	5.6	54	254	810	1,715
Pancreas	C25	12,844	7,378	15.0	237	918	2,400	3,823	5,466	10.8	168	480	1,539	3,279
Larynx	C32	9,566	8,831	18.0	247	1,419	3,504	3,661	735	1.4	32	124	276	303
Lung	C33‐34	62,608	50,802	103.5	961	5,044	16,949	27,848	11,806	23.2	526	1,635	3,569	6,076
Skin melanoma	C43	13,544	6,753	13.8	1,746	1,435	1,769	1,803	6,791	13.4	2,507	1,408	1,429	1,447
Mesothelioma	C45	2,324	1,759	3.6	48	200	582	929	565	1.1	23	79	168	295
Connective tissue	C47,49	2,898	1,596	3.3	382	296	423	495	1,302	2.6	337	228	323	414
Breast	C50	80,224	616	1.3					79,608	156.7	11,823	20,449	23,348	23,988
Cervix uteri	C53	5,125							5,125	10.1	1,600	1,257	1,153	1,115
Corpus uteri	C54	12,735							12,735	25.1	652	2,410	4,777	4,896
Ovary	C56	8,475							8,475	16.7	1,131	1,863	2,556	2,925
Prostate	C61	43,623	43,623	88.8	54	1,766	12,868	28,935						
Testicular	C62	3,421	3,421	7.0	2,772	408	162	79						
Kidney	C64‐66,68	17,552	12,125	24.7	826	1,826	4,109	5,364	5,427	10.7	435	783	1,652	2,557
Bladder	C67,D090,D303,D414	32,209	26,649	54.3	1,017	2,820	8,387	14,425	5,560	10.9	341	610	1,632	2,977
Brain	C70‐72	8,706	4,942	10.1	1,021	844	1,439	1,638	3,764	7.4	692	596	1,034	1,442
Thyroid	C73	13,089	3,249	6.6	1,082	703	779	685	9,840	19.4	3,545	2,364	2,227	1,704
Hodgkin lymphoma	C81	4,028	2,185	4.5	1,338	293	299	255	1,843	3.6	1,253	182	212	196
Non‐Hodgkin lymphoma	C82‐85,96	19,214	10,650	21.7	2,078	1,715	2,895	3,962	8,564	16.9	1,356	1,289	2,331	3,588
SLL/CLL	M9670,9823	4,932	3,058	6.2	123	347	1,018	1,570	1,874	3.7	64	247	571	992
NHL, diffuse large B	M9678‐9684	4,982	2,811	5.7	659	457	686	1,009	2,171	4.3	423	301	490	957
NHL, follicular	M9675,9690‐9698	3,116	1,493	3.0	298	313	432	450	1,623	3.2	248	282	527	566
Acute myeloid leukaemia	M9840,9861,9866‐9867,	3,377	1,804	3.7	332	248	457	767	1,573	3.1	321	222	403	627
	9870‐9874,9891‐9931													
Multiple myeloma	M9731‐9734	6,669	3,536	7.2	164	468	1,125	1,779	3,133	6.2	132	396	929	1,676
All types	C00‐43,45‐96, D090,D303,D414	508,617	281,687	573.7	19,400	32,524	87,862	141,901	226,930	446.8	30,425	43,823	65,414	87,268

Cancer Registries with ≥18 years of observation, age 15‐74 years; Cancer type or subtype with >2000 cases in the period and areas covered by CRs.

The observed relative survival (RS) was calculated for adult cases (aged 15‐74 years) diagnosed in 1985‐2011 and followed‐up until 2013, using the cohort method and the Ederer II approach.^4^, ^12^

Indicators of long‐term survival and cancer cure were obtained by applying, for each combination of cancer type and sex, mixture cure models to RS data, stratified by age groups (15‐44, 45‐54, 55‐64, 65‐74 years), and three‐year diagnostic periods (ie, 1985‐1987, 1988‐1990..., 2009‐2011).^18^, ^19^ Only cancer types (first two ICD10 digits, or ICDO3 groups for hemopoietic neoplasms) with at least 2 000 adult cases recorded in the Italian registries in 1985‐2011 were considered (Table 1).

The three indicators of long‐term survival and cancer cure for Italian patients were defined as follows: (a) median life expectancy of fatal tumors (LEFT), reached when 50% of patients with a fatal tumor had died^18^; (b) the proportion of cancer patients expected to reach the same death rates of the general population of the same sex and age (cure fraction, CF)^4^, ^18^, ^20^; and (c) the number of years after cancer diagnosis necessary to eliminate, or at least to make negligible, the excess mortality due to cancer (time to cure, TTC).^4^, ^6^

From a statistical and epidemiological point of view, CF is reached when the cancer specific excess mortality approaches zero and patients will die of causes other than that neoplasm.^21^ Given the conditional relative survival (CRS) as the survival experience of the cohort alive *n* years after the diagnosis of a specific cancer, the TTC was measured as the number of years necessary for model‐based 5‐year CRS to reach 90% or 95%, two clinically or epidemiologically relevant thresholds of fading cancer excess mortality.^4^, ^6^

For most cancer types, a Weibull distribution was used to model the excess mortality function for fatal cases (ie, those who will never reach the same death rates of the general population). For breast cancer in women, bladder and thyroid cancers, and Hodgkin lymphoma a better fit was obtained by using an exponential distribution. All models were stratified by age, assuming linearity in the period of diagnosis effects.^18^ Changes of LEFT and CF over time were calculated accordingly for two periods of diagnosis (1990 and 2000).

The goodness of fit of model‐based RS was evaluated using the likelihood ratio test and cure models converged for every cancer type and sex. In addition, a comparison of observed and model‐based RS and 5‐year CRS was conducted^22^ until 25 years after diagnosis for all cancer types, sex, age groups, and period of diagnosis (Appendices S1 and S2). The model fitting to observed RS was good with few exceptions for cancer types with a very poor long‐term survival. For these cancers, inconsistencies between the observed and model‐based CRS emerged. Therefore, TTC were presented only for cancer types with a CF >20% in men or women.

## 3 RESULTS

More than half a million Italian cancer patients aged 15‐74 years in 1985‐2011 (281 687 men and 226 930 women) contributed to the study (Table 1), 79 608 women with breast cancer, 62 608 cases with lung, 43 623 with prostate, and 41 089 with colon cancers.

In 1990 and in both sexes, the median LEFT was less than half a year for patients with liver, gallbladder, pancreas, lung, brain cancers, and with acute myeloid leukemias (Table 2). Conversely, a median LEFT longer than five years was estimated ‐in 1990‐ for cancers of the larynx (>10 years), breast (8.1), prostate (5.2), and follicular lymphomas (6.6). They were 5.8 years for testicular cancer, and 6.3 years in men and 6.9 years in women with Hodgkin lymphoma diagnosed below age 45 years (ie, the vast majority of cases).

**Table 2:** Median life expectancy of fatal tumors by cancer type, sex, and period. Italy

Year of diagnosis	MEN				WOMEN				
Cancer type	1990	2000	Variation		1990	2000		Variation	
	years	years	years	%	years	years		years	%
Oral cavity and pharynx	1.9	2.2	0.36	19%	3.8		4.2	0.42	11%
Esophagus	0.6	0.7	0.15	26%	0.7		0.8	0.13	19%
Stomach	0.6	0.7	0.11	17%	0.7		0.8	0.06	9%
Colon	1.4	1.6	0.14	10%	1.5		1.6	0.12	8%
Rectum	2.0	2.2	0.21	10%	1.9		2.1	0.19	10%
Liver	0.4	0.7	0.32	80%	0.5		0.8	0.34	69%
Gallbladder	0.4	0.6	0.12	27%	0.4		0.4	0.10	27%
Pancreas	0.3	0.4	0.09	29%	0.4		0.5	0.11	29%
Larynx	10.9	10.8	−0.10	−1%	10.1		13.6	3.50	35%
Lung	0.5	0.6	0.09	16%	0.5		0.7	0.16	29%
Skin melanoma	2.5	2.8	0.33	13%	4.3		4.6	0.27	6%
Mesothelioma	0.9	1.0	0.12	13%	1.0		1.1	0.09	10%
Connective tissue	2.2	2.4	0.13	6%	2.0		2.1	0.15	8%
Breast^b^					8.1		9.4	1.38	17%
Cervix uteri					2.4		2.5	0.09	4%
Corpus uteri					3.9		4.0	0.10	3%
Ovary					1.8		2.0	0.23	13%
Prostate	5.2	7.4	2.21	42%					
Testicular^a^	5.8	6.1	0.22	4%					
Kidney	8.5	>15	–	–	3.2	4.2		0.96	30%
Bladder^b^	4.3	4.7	0.44	10%	3.3	3.5		0.21	6%
Brain	0.5	0.6	0.11	24%	0.4	0.6		0.14	31%
Thyroid^a^	3.6	3.7	0.08	2%	4.6	4.6		0.02	0%
Hodgkin lymphoma^a^	6.3	6.6	0.30	5%	6.9	7.5		0.61	9%
Non‐Hodgkin lymphoma	3.1	5.0	1.94	63%	5.4	9.7		4.35	81%
SLL/CLL^c^	3.6	3.6	0.04	1%	5.1	4.9		−0.21	−4%
NHL, diffuse large B	0.9	1.2	0.27	29%	1.8	2.1		0.38	22%
NHL, follicular	6.6	11.8	5.25	80%	6.6	12.90		6.28	95%
Acute myeloid leukaemia	0.3	0.5	0.18	40%	0.3	0.5		1.01	28%
Multiple myeloma	3.0	4.2	1.19	66%	3.7	4.7		0.19	57%
All types	1.0	1.4	0.41	40%	2.3	2.7		0.46	20%

Calculated as the median (50th percentile) relative survival, estimated through the best fitting model‐based distributions. Patients aged 15‐74 years, except when specified.

a Patients aged 15‐44 years.

b Patients aged 65‐74 years.

c Patients aged 55‐74 years.

Between 1990 and 2000, the median LEFT increased by more than one year for patients with breast (from 8.1 to 9.4 years) or prostate cancer (from 5.2 to 7.4 years), and ‐in both sexes‐ non‐Hodgkin lymphoma (NHL), in particular follicular NHL. Conversely, a limited (ie, <2 months) increase was estimated for stomach, colon, gallbladder, pancreas, lung, cervix and corpus uteri, brain, thyroid cancers and small lymphocytic lymphoma/chronic lymphocytic leukemia (SLL/CLL). For most cancer types, the median LEFT slightly decreased with age (Appendix S3).

**Figure 1:**
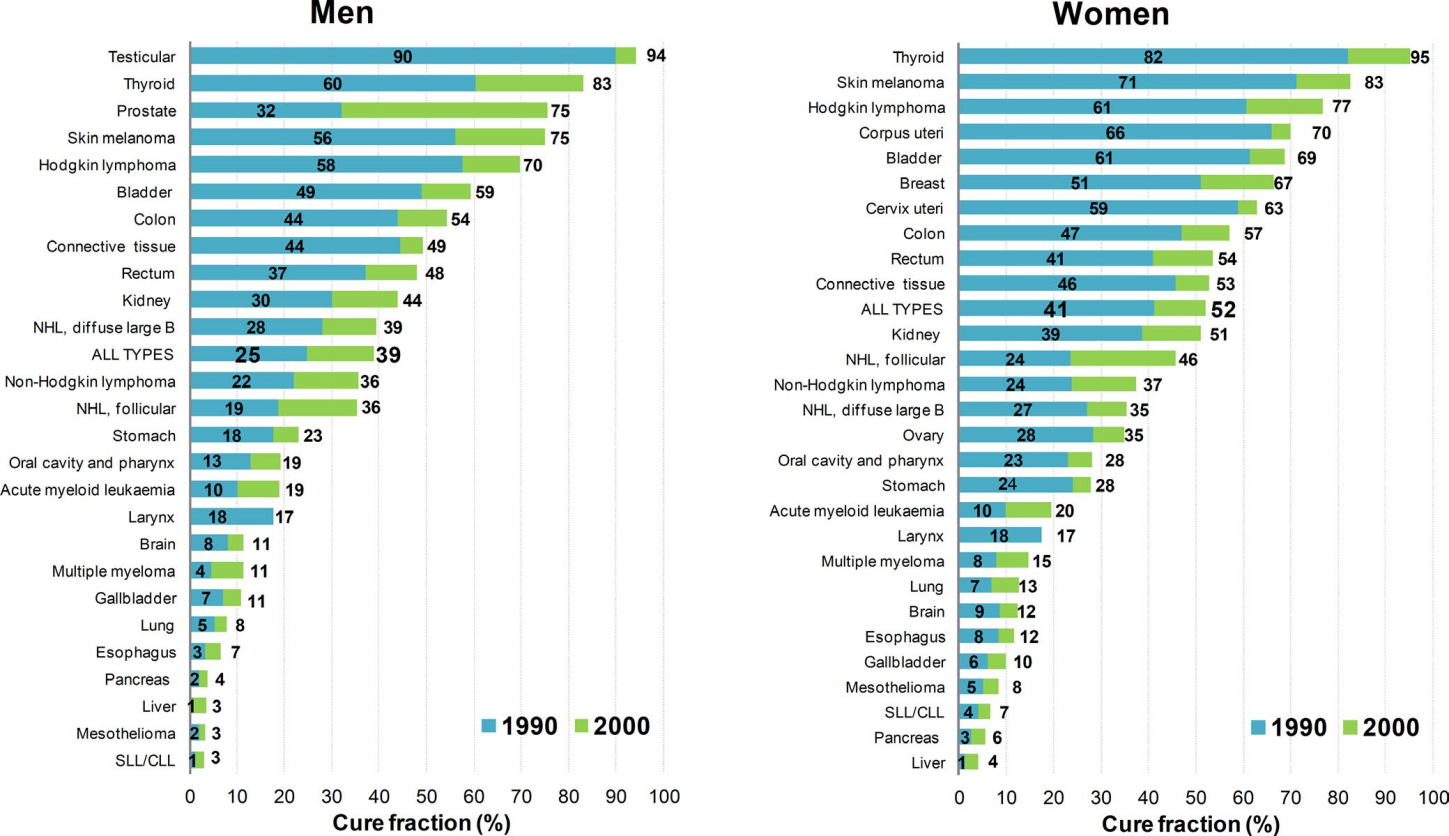
Estimated cure fraction (%)^a^ by sex, cancer type, and year of diagnosis in Italy. ^a^Calculated as weighted means of corresponding cure fractions for the four age groups (Appendix S4) with weights the number of incident cases. Patients aged 15‐74 years

For cancer cases diagnosed in 2000, the CF was 39% for the combination of all cancer types (ie, weighted by the number of cases by type and age) in men (Figure 1). CF was >50% for patients with testicular cancers (94%), thyroid (83%), prostate (75%), skin melanoma (75%), Hodgkin lymphoma (70%), bladder (59%) or colon cancers (54%). In women, the CF for all cancers was 52%, the highest CF was estimated for thyroid cancer (95%), skin melanoma (83%), Hodgkin lymphoma (77%), corpus uteri (70%), bladder (69%), and breast cancer (67%). Conversely, for cases diagnosed until 2000, a CF <10% was estimated in both sexes for cancers of the liver and pancreas, mesothelioma, and SLL/CLL. The CF increased approximately 10 percentage points between 1990 and 2000 for most common cancer types (ie, colon and rectum, breast or bladder cancer), and across most age groups (Appendix S4). Notably, a nearly doubled CF emerged in Italy for prostate cancers between 1990 and 2000, while only a limited (<5%) increase was observed for the other most fatal cancer types. A marked CF decrease emerged with age (Appendix S4).

Table 3 shows the observed number of patients alive at 10 years since diagnosis, the observed 5‐year CRS for patients alive 10 years after diagnosis, and the estimated number of years necessary for model‐based 5‐year CRS to exceed 90% or >95%. Men and women who already survived 10 years showed an observed 5‐year CRS >95% in all age groups when the diagnoses were colon cancer, skin melanoma, testicular and thyroid cancers (94% in 65‐74 years men). Patients with these cancer types indeed reached 5‐year CRS >95% in less than 10 years (Table 3). Notably, TTC (with 95% threshold for 5‐year CRS) was reached in less than 1 year for thyroid cancer patients below the age of 55 years. Conversely, a small but non negligible excess risk of death was still present even after 15 years since diagnosis for women with breast cancer and men with prostate and bladder cancers. In both sexes, a clear long‐term excess risk of death emerged for most smoking‐related cancers (Appendices S1 and S2), and for hemolymphopoietic neoplasms, except for Hodgkin lymphoma (Table 3).

**Table 3:** Indicators of time to cure^a^ by sex, cancer type^b^, and age in Italy

Age (y)	15‐44				45‐54				55‐64				65‐74			
	Observed		Years to 5‐y CRS^c^		Observed		Years to 5‐y CRS^c^		Observed		Years to 5‐y CRS^c^		Observed		Years to 5‐y CRS^c^	
SEX, Cancer type	At 10 y	5/10 CRS	>90%	>95%	At 10 y	5/10 CRS	>90%	>95%	At 10 y	5/10 CRS	>90%	>95%	At 10 y	5/10 CRS	>90%	>95%
MEN																
Oral cavity and pharynx	247	85%	13	18	485	82%	15	20	615	79%	16	>20	343	76%	17	>20
Stomach	163	97%	6	9	360	97%	6	8	658	97%	7	9	701	89%	11	16
Colon	349	99%	5	7	913	99%	5	7	2,204	97%	6	8	2,725	97%	6	9
Rectum	148	96%	7	9	476	98%	6	8	1,059	94%	8	11	1,177	94%	8	11
Skin melanoma	820	99%	4	6	597	96%	5	9	643	98%	5	8	472	98%	6	8
Connective tissue	158	96%	6	10	127	94%	7	12	134	93%	9	13	90	91%	10	14
Prostate	17	93%	6	14	575	92%	7	15	4,636	92%	8	16	8,719	91%	9	17
Testicular	1,601	99%	<1	<1	251	100%	<1	<1	89	98%	1	1	23	94%	9	11
Kidney	398	96%	2	7	762	93%	6	17	1,481	91%	9	>20	1,294	88%	14	>20
Bladder	630	98%	<1	1	1,605	95%	3	11	3,644	92%	9	16	4,347	90%	11	16
Thyroid	506	100%	<1	<1	276	96%	<1	7	255	97%	6	9	145	94%	8	11
Hodgkin lymphoma	792	97%	0	7	130	93%	7	15	101	80%	>20	>20	52	86%	12	16
Non‐Hodgkin lymphoma	889	91%	8	>20	678	89%	12	>20	851	87%	15	>20	742	85%	19	>20
NHL, diffuse large B	235	99%	4	6	151	95%	7	10	156	88%	12	>20	141	88%	12	19
NHL, follicular	160	92%	3	>20	134	88%	20	>20	137	83%	>20	>20	100	76%	>20	>20
WOMEN																
Oral cavity and pharynx	159	89%	12	>20	179	85%	16	>20	227	83%	19	>20	263	80%	>20	>20
Stomach	120	99%	5	6	215	97%	7	9	430	97%	6	8	574	93%	9	13
Colon	344	100%	5	6	893	98%	5	7	1,993	98%	5	7	2,593	97%	6	8
Rectum	125	99%	5	7	363	97%	7	9	757	98%	6	8	880	96%	7	10
Skin melanoma	1,281	97%	<1	6	703	98%	2	6	697	96%	2	8	575	95%	6	10
Connective tissue	141	97%	5	9	89	96%	7	10	125	95%	7	11	92	94%	8	12
Breast	5,785	92%	9	14	10,663	94%	5	12	11,539	92%	8	17	9,712	89%	12	>20
Cervix uteri	858	99%	3	6	589	100%	4	6	511	93%	8	13	349	90%	11	16
Corpus uteri	355	96%	1	7	1,443	99%	1	4	2,395	95%	3	11	1,842	94%	6	11
Ovary	544	97%	5	9	558	94%	9	11	558	91%	10	13	362	93%	9	12
Kidney	214	100%	3	5	365	95%	4	11	684	92%	7	19	779	91%	10	>20
Bladder	218	100%	<1	2	330	99%	2	4	821	95%	5	10	1,126	94%	8	11
Thyroid	1,803	100%	<1	<1	1,111	99%	<1	<1	937	99%	<1	3	560	98%	4	6
Hodgkin lymphoma	750	97%	<1	4	104	95%	<1	11	94	85%	18	>20	55	77%	20	>20
Non‐Hodgkin lymphoma	644	93%	4	17	590	91%	9	>20	897	88%	15	>20	889	85%	>20	>20
NHL, diffuse large B	160	98%	4	7	110	92%	7	20	139	87%	14	>20	166	83%	17	>20
NHL, follicular	128	95%	1	13	138	91%	9	>20	198	87%	20	>20	163	81%	>20	>20

At 10 years = Patients alive at 10 years since diagnosis; CRS, Conditional Relative Survival.

a Patients alive at 10 years since diagnosis (At 10 years), observed 5‐year Conditional Relative Survival for patients alive 10 years after diagnosis (5/10 CRS), and years to reach 5‐year Conditional Relative Survival (5‐year CRS) >90% or >95%.

b Cancer types with CF >20% in men or women.

c Model‐based 5‐year CRS centered in 1995.

**Figure 2:**
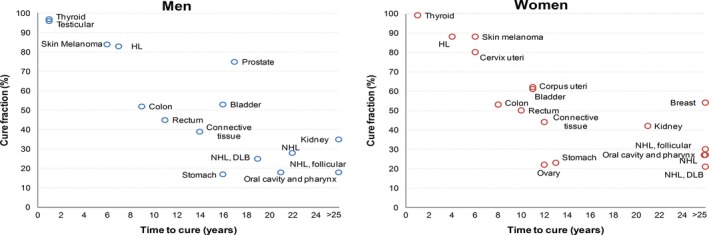
Cure fraction (%) and time to cure^a^ by sex for selected cancer types^b ^in Italy. ^a^Calculated in 2000 for the most frequent age group (Table 3 and Appendix S4), that is, 65‐74 years but Connective Tissue, Cervix uteri, Testis, Thyroid, and Hodgkin lymphoma (15‐44 years). ^b^Cancer types with CF >20% in men or women

Figure 2 shows CF and TTC for major cancer types with CF>20%. Major patterns of cancer types emerged. The first included cancers with a CF >80% and a TTC ≤5‐6 years (eg, testicular, thyroid, skin melanoma); the second, cancers with a CF of approximately 50% and a TTC <10 years (colon, rectum); the third, cancers showing a CF of approximately 50% and TTC >10 years (breast, prostate, and bladder). In addition, the most severe pattern included some cancer types with a CF <20% (Figure 1) and uncertain TTC.

## 4 DISCUSSION

This study provided updated estimates of three relevant indicators of long‐term survival and cancer cure for Italian patients, which are still lacking in current cancer statistics.

The median LEFT increased 4‐5 months in both sexes between 1990 and 2000, with noteworthy variations across cancer types. Patients who died of prostate cancer had 2‐year longer median LEFT when diagnosed in 2000 (7.4 years), as compared to those diagnosed in 1990 (5.2). A similar increase emerged for breast cancer (from 8.1 to 9.4), while the median LEFT showed a small increase (<2 months) for colon, rectal or lung cancers. In the same decade, an approximately 10% increase in CF was estimated for adult patients and, notably this increase was consistent across cancer types.

Our results, along with the findings from similar studies,^5^, ^6^, ^7^, ^8^, ^9^, ^10^ supported the characterization of four major patterns of cancer types which may correspond to specific follow‐up strategies. The less severe characterization included cancer types with 5‐year RS and CF >80% (eg, testicular and thyroid cancers below age 55 years). For these good prognosis cancers less intense surveillance scheme may be warranted, because after one or two years since diagnosis no relevant excess mortality remained.^6^, ^9^, ^10^ Excess mortality became negligible in less than 10 years for patients below age 45 years with Hodgkin lymphoma, skin melanoma, and cervical cancer.^6^, ^23^

A second group included cancer types with negligible excess risk of death within 10 years from diagnosis (eg, colorectal and younger patients with stomach cancer). For these cancers, however, only medium or low CFs have been reported (approximately 50% and 20%, respectively).^5^, ^6^, ^9^, ^10^

In the third group, including women with breast cancer, despite dramatic improvements in survival over the past decades, a small (ie, 5‐year CRS <10%) but persistent (for >15 years) increased death risk was observed.^8^, ^10^, ^20^, ^24^, ^25^, ^26^ It should be noted, however, that less than half the women with breast cancer will die because of that cancer in the first 20 years following diagnosis (ie, 20‐year RS >50%, Appendix S2).^27^, ^28^ A very similar pattern emerged for patients with prostate and bladder cancers, for which a long follow‐up is needed.

The last group included cancer types showing 5‐year RS <20% (eg, lung or pancreatic cancer),^5^, ^7^, ^9^, ^10^ whose patients in this group may hardly reach the same expected death rate of the general population, because of the severe prognoses of these specific neoplasms, and the competitive risks associated with lifestyle risk factors. In particular, a vast majority of respiratory tract cancer patients are smokers who carry a high risk of smoking‐related mortality.^29^ A similar excess of noncancer related mortality is expected for people infected with hepatitis C virus, a large proportion of liver cancer patients. These effects could result in reduced long‐term RS and in prolonged or indefinite TTC.

### 4.1 Strengths and limitations

To the best of our knowledge, this is the first study reporting trends of LEFT and CF for a large number of cancer types. In addition, in this study a careful validation was conducted by comparing observed RS, 5‐years CRS, and the corresponding model‐based curves by follow‐up time to 25 years after diagnosis (Appendices S1 and S2). The accuracy of the present estimates relied on the length of follow‐up and size of the study population. The follow‐up period appeared long enough to provide reliable estimates of median LEFT, CF, and TTC for several cancer types.^30^ Indeed, our survival estimates were based on a very large population‐based cohort of patients, which allowed estimates by some relevant histological subtype (ie, diffuse large‐B‐cell and follicular NHL, and SLL/CLL or acute myeloid leukemias).

A first limitation is related to the representativeness of our results at the national level, since the long‐established cancer registries contributing to this study cover only 10% of the Italian population. Variability across regions of present indicators cannot be excluded, although cancer registries were well distributed across all Italian areas.^11^ Moreover, the generalization of results to other countries, herein presented, is also uncertain albeit the Italian survival levels were similar to those of most central and southern European countries.^12^ Additional limitations are related to the use of cure models.^22^, ^31^ These models are influenced by the choice of the survival distribution of fatal tumors. Most importantly, the estimates are critical for cancers maintining a long‐term excess mortality risk because the follow‐up period available may not be sufficient to observe the deaths of all fatal cases, ie the plateau in the survival curve. This means that there might have been an identifiability issue of the CF and LEFT.^5^, ^22^ Moreover, it should be noted that the estimation of TTC is sensible to the choice of the CRS threshold and to the use of different definitions,^4^, ^6^, ^10^ in particular for prostate or breast cancer in women. To take into account these critical points, we validated all models in addition to provide observed 5‐year CRS estimates at a fixed time point (10 years after diagnosis) and of TTC at different thresholds (ie, 90% and 95%).^4^, ^6^ Finally, information on other important prognostic factors (ie, stage and treatment) is not routinely collected by Italian CRs, and population‐based studies with a long follow‐up can hardly allow fine stratifications to assess long‐term survival, or cure of small subgroups of patients exposed to fast‐changing therapies.

## 5 CONCLUSIONS

Our study confirmed, between 1990 and 2000 a general improvement of prognosis and cure of adult Italian cancer patients, measured here in terms of cure fraction and of life expectancy of fatal cases. The findings highlighted a 10% average increase of cure fraction for adult Italian cancer patients, and a 4‐5 months increase of median LEFT in a decade. Excess cancer mortality risk disappeared in <10 years for testis, thyroid, colon cancers, and melanoma, while it remained for >15 years for breast and prostate cancers.

Detailed (eg, by sex, age, year of diagnosis) estimates of different indicators of long‐term survival and cancer cure are useful to health professionals in enhancing the efficacy of long‐term follow‐up, a goal which can be reached through appropriate tools and procedures.^14^, ^32^, ^33^

In conclusion, recognizing a cancer patient as cured, and quantifying his/her long‐term excess risk of death, represents a valuable opportunity to improve his/her quality of life^34^, ^35^ and social or professional perspectives, as well.^8^

## ETHICS APPROVAL AND CONSENT TO PARTICIPATE

The Italian legislation identifies Cancer Registries as collectors of personal data for surveillance purposes without explicit individual consent. The approval of a research ethics committee was not required, since this study is a descriptive analysis of individual data without any direct or indirect intervention on patients (Decreto del Presidente del Consiglio dei Ministri, 3/3/2017, Identificazione dei sistemi di sorveglianza e dei registri di mortalità, di tumori e di altre patologie, 17A03142, GU Serie Generale n.109 del 12‐05‐2017 (Available at: http://www.gazzettaufficiale.it/eli/id/2017/05/12/17A03142/sg, last access: 10/10/2018).

## DISCLOSURE

The authors have declared no conflicts of interest.

## AUTHOR'S CONTRIBUTIONS

Luigino Dal Maso and Stefano Guzzinati drafted the study protocol, designed the study, and drafted the manuscript with the support of Roberta De Angelis. All authors (Luigino Dal Maso, Chiara Panato, Stefano Guzzinati, Diego Serraino, Silvia Francisci, Laura Botta, Riccardo Capocaccia, Andrea Tavilla, Anna Gigli, Emanuele Crocetti, Massimo Rugge, Giovanna Tagliabue, Rosa Angela Filiberti, Giuliano Carrozzi, Maria Michiara, Stefano Ferretti, Rosaria Cesaraccio, Rosario Tumino, Fabio Falcini, Fabrizio Stracci, Antonietta Torrisi, Guido Mazzoleni, Mario Fusco, Stefano Rosso, Francesco Tisano, Anna Clara Fanetti, Giovanna Maria Sini, Carlotta Buzzoni, Roberta De Angelis) and the AIRTUM Working Group revised the study protocol, collected data, prepared raw data for the study database, and corrected data after quality controls. Chiara Panato did the statistical analyses with the support of Stefano Guzzinati, Laura Botta, Andrea Tavilla, and Luigino Dal Maso. Diego Serraino, Silvia Francisci, Riccardo Capocaccia, Anna Gigli, Emanuele Crocetti, and Roberta De Angelis specifically supported Luigino Dal Maso in the interpretation and and discussion of clinical implication of study results. All authors revised the preliminary results and the report, and contributed to data interpretation, report writing, and reviewed and approved the final version.

## DATA AVAILABILITY STATEMENT

Dataset supporting our findings is available, according to AIRTUM guidelines, at the following website: www.registri-tumori.it.

## APPENDIX 1

### AIRTUM Working group

Saverio Virdone (CRO Aviano), Gemma Gatta (INT Milan), Manuel Zorzi (Veneto Cancer Registry‐CR), Sandra Mallone (ISS Rome), Federica Toffolutti (Friuli Venezia Giulia CR), Antonio Giampiero Russo (Milano CR), Anna Luisa Caiazzo (Salerno CR), Lucia Mangone (Reggio Emilia CR), Walter Mazzucco (CR of Palermo and province), Fabio Pannozzo (Latina CR), Paolo Ricci (Mantova CR), Gemma Gola (Como CR), Giuseppa Candela (Trapani CR), Antonella Sutera Sardo (Catanzaro CR).
