# Supplementary material for: Prognosis and cure of long‐term cancer survivors: A population‐based estimation
Source: Cancer Med. 2019 Jun 17;8(9):4497–507. doi: 10.1002/cam4.2276 (PMC6675712; doi:10.1002/cam4.2276)
Supplement: Supplementary file 1 [file CAM4-8-4497-s001.pdf]

Appendix 1. Observed and model-based 25-year relative survival (RS) and 5-year conditional relative survival (CRS) by cancer type in the most frequent age group[a] in men. Italy, 1985-2011.

Observed RS — 95% CI — Observed 5-yr CRS
Model-based RS — Model-based 5-yr CRS

Oral cavity and pharynx[a]

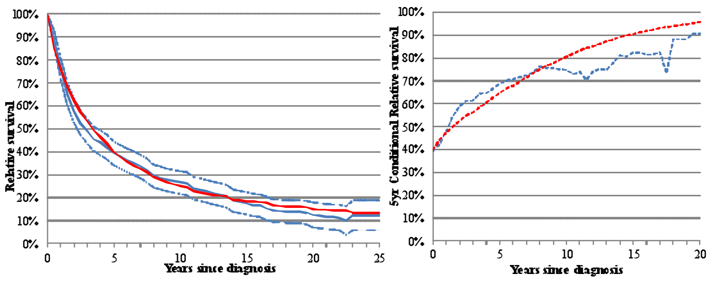

Cases contributing to estimates
3552 1286 615 245 65 13

Esophagus

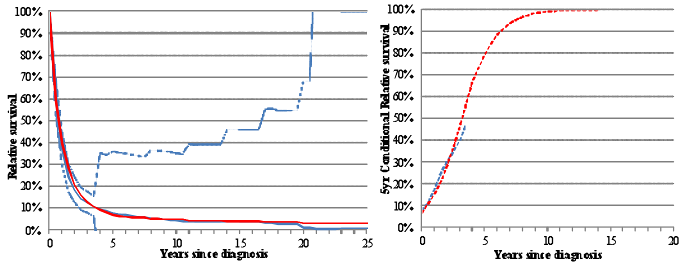

Cases contributing to estimates
2002 188 77 34 3 2

Stomach

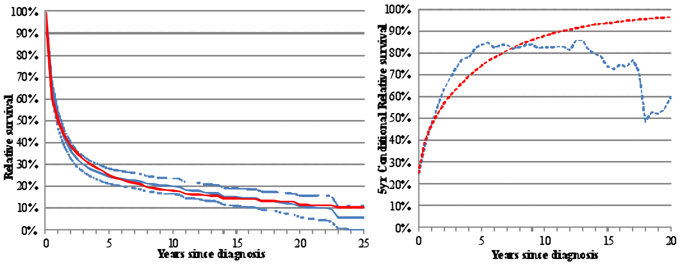

Cases contributing to estimates
6732 1386 701 259 58 3

Colon

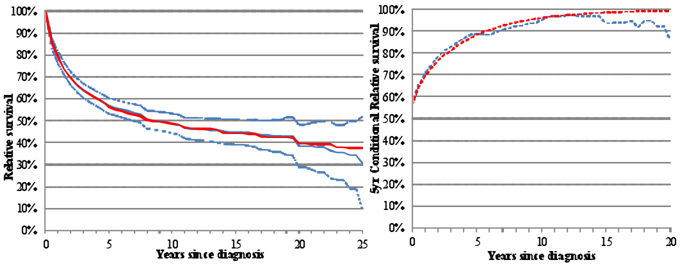

Cases contributing to estimates
12313 5808 2725 1083 209 16

Rectum

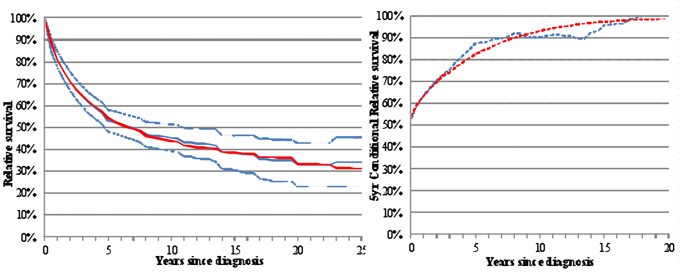

Cases contributing to estimates
5451 2436 1177 435 91 13

Liver

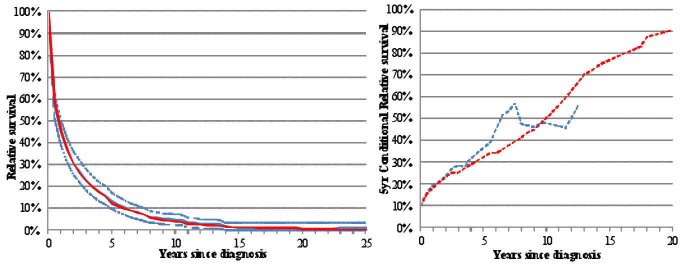

Cases contributing to estimates
2462 347 69 11 3 1

Gallbladder

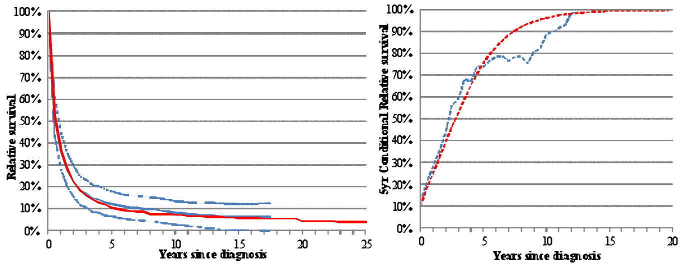

Cases contributing to estimates
1306 146 61 18 5 2

Pancreas

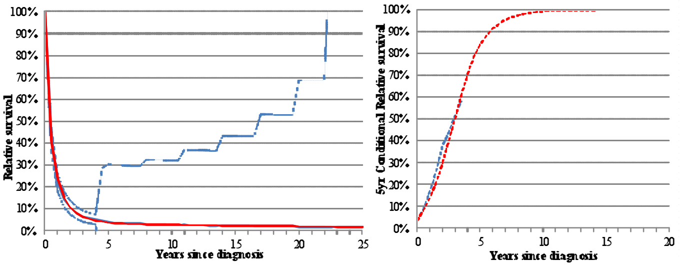

Cases contributing to estimates
3737 146 54 25 8 2

[a] 65-74 years but Oral cavity and pharynx (55-64 years), Testicular, Thyroid and Hodgkin lymphoma (15-44 years) and SLL/CLL (55-74 years).

Continues (Appendix 1)

Appendix 1. Continued...

Larynx

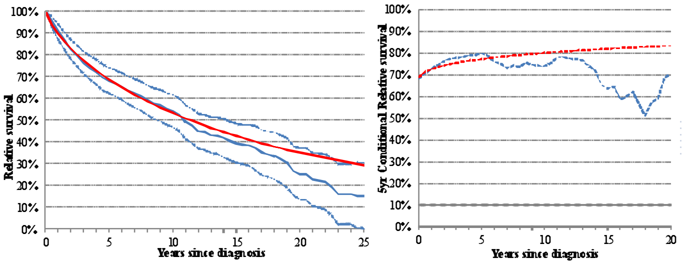

Cases contributing to estimates

3633 2058 1015 365 67 4

Prostate

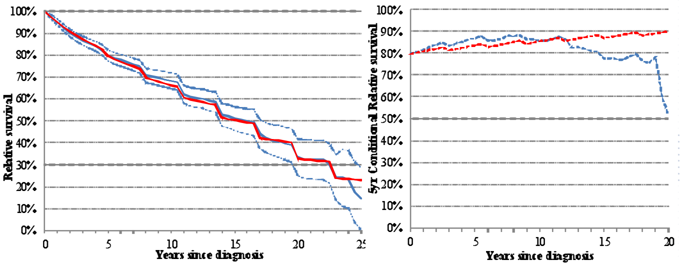

Cases contributing to estimates

28825 19527 8719 2180 266 9

Lung

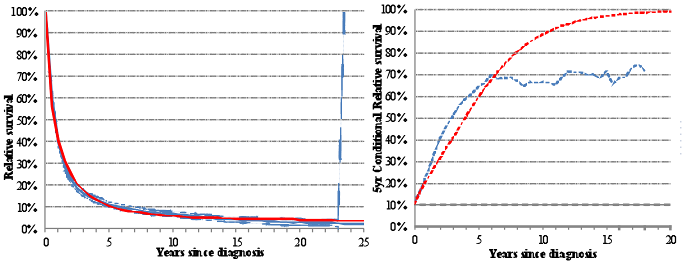

Cases contributing to estimates

27348 2766 1008 310 61 5

Testicular[a]

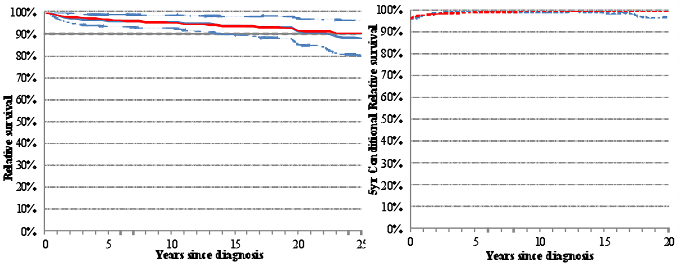

Cases contributing to estimates

2768 2325 1601 927 395 94

Skin melanoma

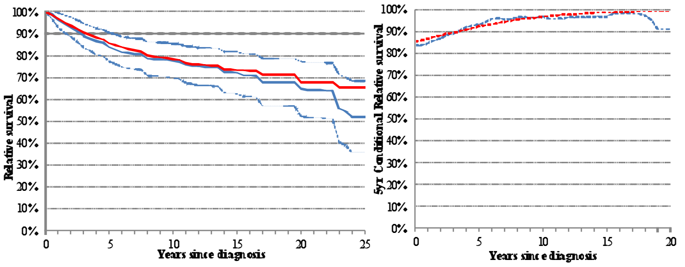

Cases contributing to estimates

1745 1316 820 458 160 23

Kidney

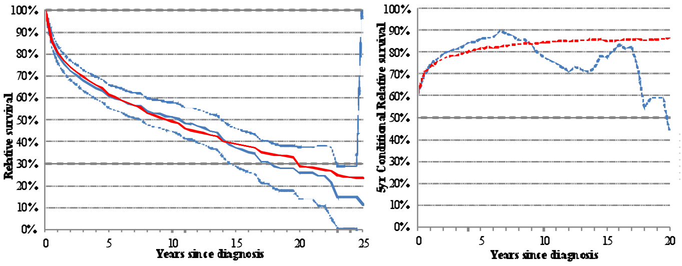

Cases contributing to estimates

5286 2695 1294 431 74 3

Mesothelioma

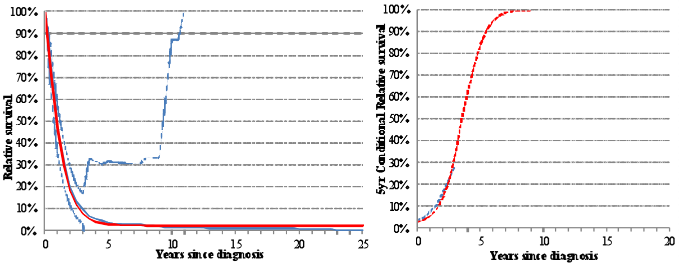

Cases contributing to estimates

926 33 13 7 4 3

Bladder

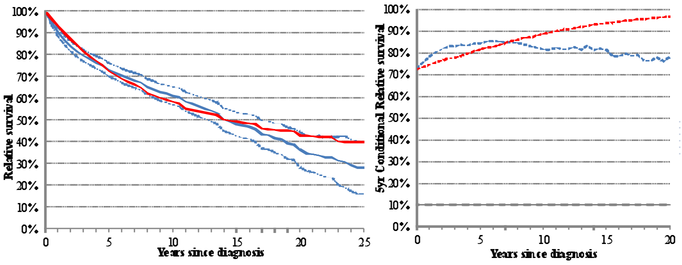

Cases contributing to estimates

14363 8564 4347 1570 279 22

Connective tissue

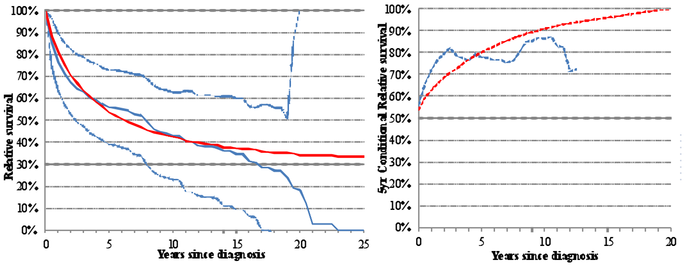

Cases contributing to estimates

492 214 90 31 5 2

Brain

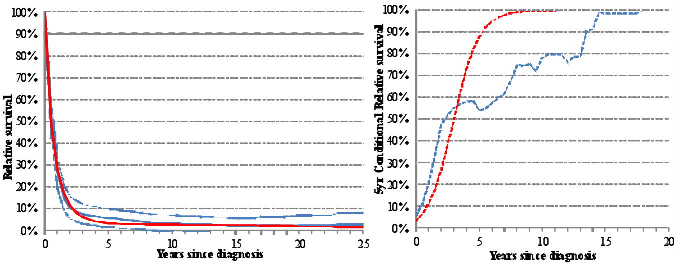

Cases contributing to estimates

1607 71 23 7 3 2

[a] 65-74 years but Oral cavity and pharynx (55-64 years), Testicular, Thyroid and Hodgkin lymphoma (15-44 years) and SLL/CLL (55-74 years).

Continues (Appendix 1)

Appendix 1. Continued...

Thyroid[a]

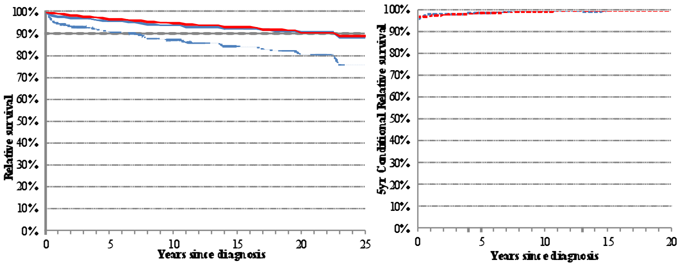

Cases contributing to estimates
1082 877 506 276 117 30

Non-Hodgkin lymphoma, DLBC

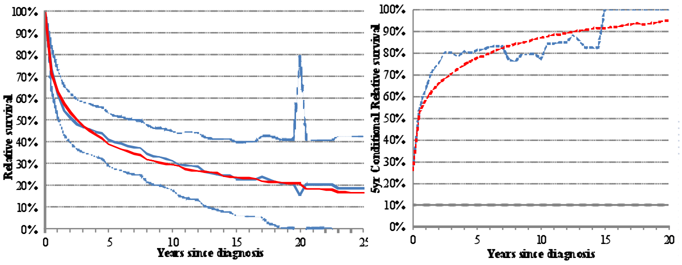

Cases contributing to estimates
1003 346 141 45 14 3

Hodgkin lymphoma[a]

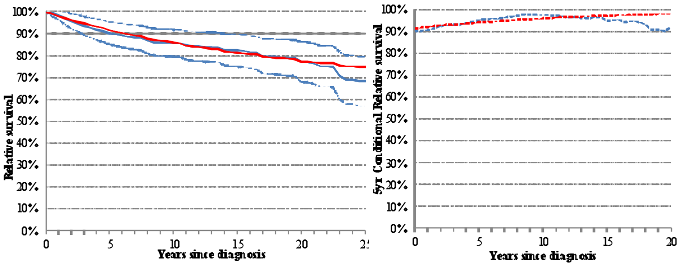

Cases contributing to estimates
1335 1098 792 498 237 59

Non-Hodgkin lymphoma, Follicular

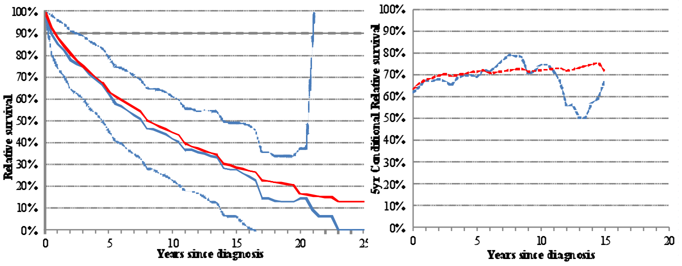

Cases contributing to estimates
449 244 100 28 5 2

Non-Hodgkin lymphoma

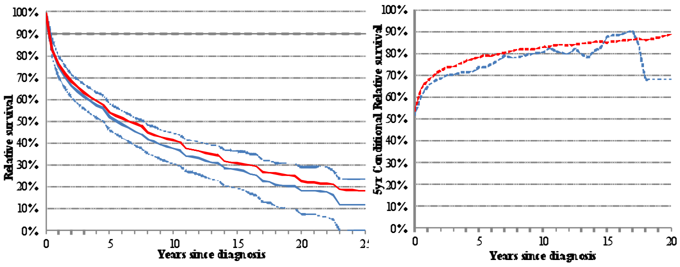

Cases contributing to estimates
3936 1755 742 241 39 6

Acute myeloid leukemia

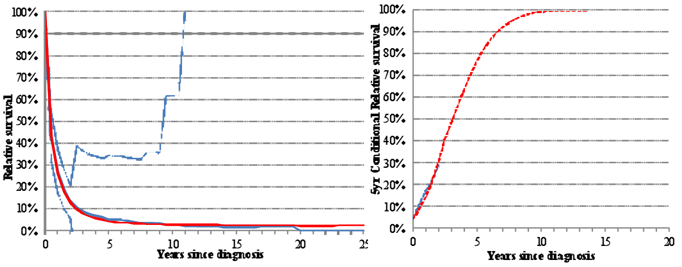

Cases contributing to estimates
757 36 14 8 3 2

Non-Hodgkin lymphoma, SLL/CLL[a]

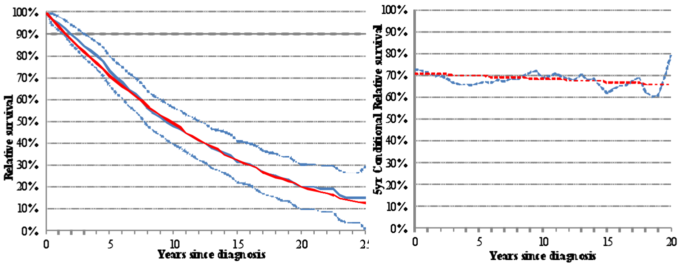

Cases contributing to estimates
2580 1623 704 236 50 6

Multiple myeloma

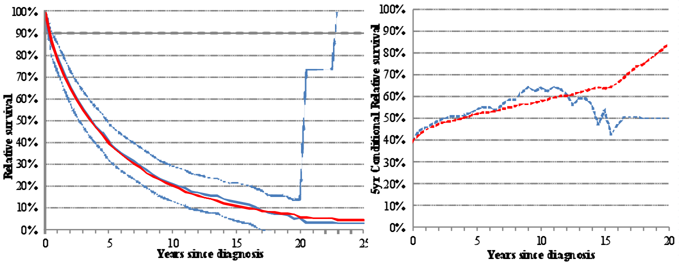

Cases contributing to estimates
1762 593 191 47 5 2

[a] 65-74 years but Oral cavity and pharynx (55-64 years), Testicular, Thyroid and Hodgkin lymphoma (15-44 years) and SLL/CLL (55-74 years).

Appendix 2. Observed and model-based 25-year relative survival (RS) and 5-year conditional relative survival (CRS) by cancer type in the most frequent age group[a] in women. Italy, 1985-2011.

Observed RS — 95% CI — Observed 5-yr CRS
Model-based RS — Model-based 5-yr CRS

Oral cavity and pharynx

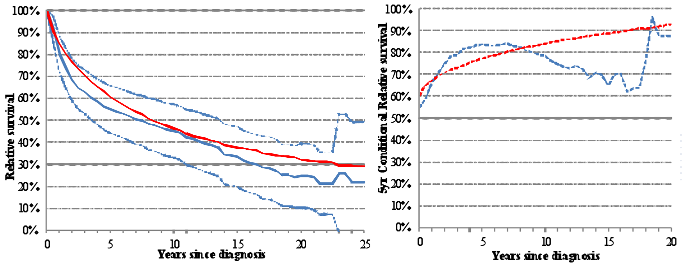

Cases contributing to estimates

1040 500 263 92 20 5

Liver

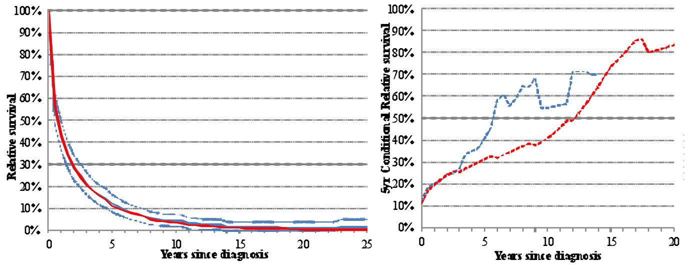

Cases contributing to estimates

2517 349 70 12 4 2

Esophagus

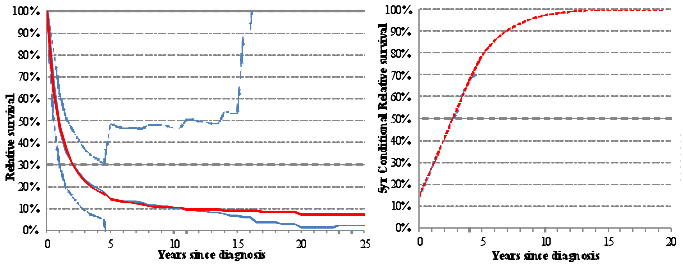

Cases contributing to estimates

374 55 29 11 3 2

Gallbladder

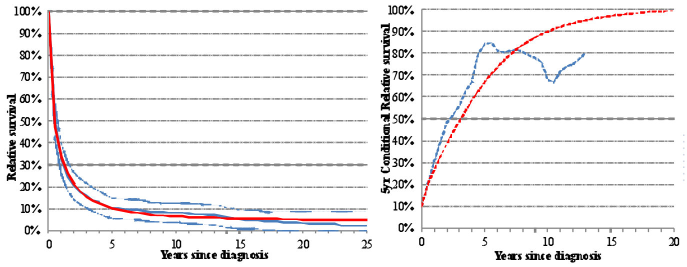

Cases contributing to estimates

1690 164 89 31 10 2

Stomach

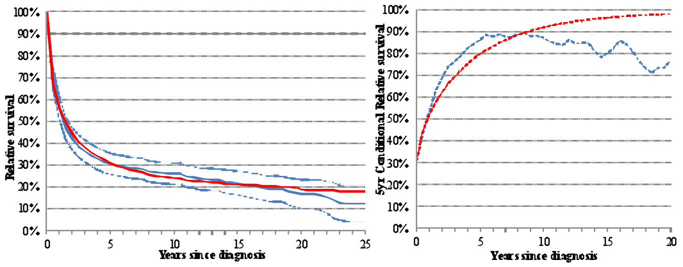

Cases contributing to estimates

3578 978 574 283 78 10

Pancreas

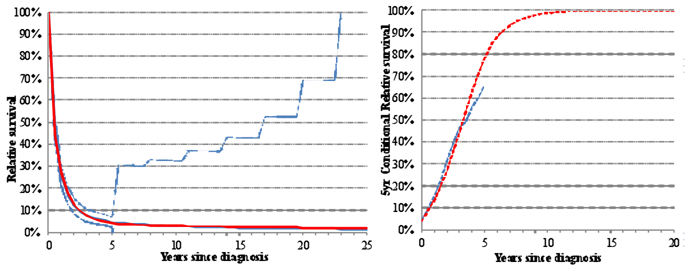

Cases contributing to estimates

3185 152 57 27 8 2

Colon

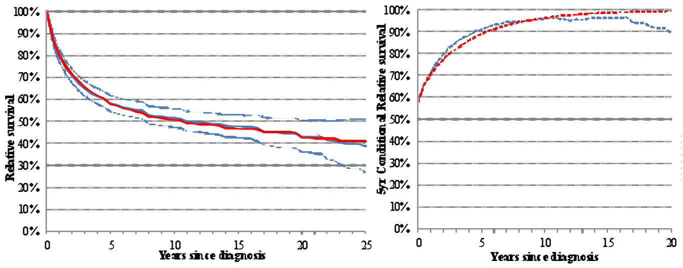

Cases contributing to estimates

8943 4594 2593 1238 325 33

Larynx

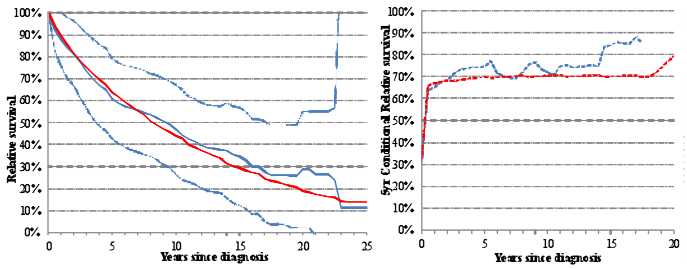

Cases contributing to estimates

300 171 100 39 12 2

Rectum

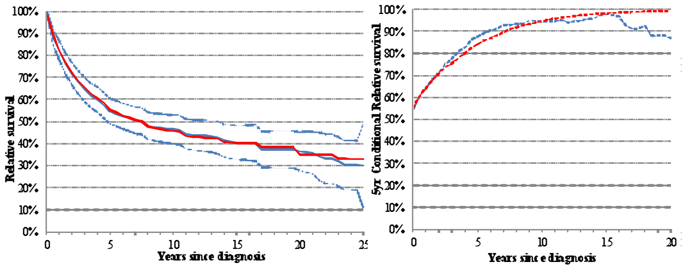

Cases contributing to estimates

3221 1600 880 406 122 15

Lung

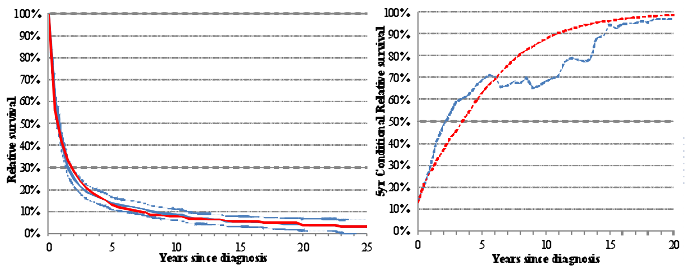

Cases contributing to estimates

5939 807 323 110 25 3

[a] 65-74 years but Skin melanoma, Cervix uteri, Thyroid and Hodgkin lymphoma (15-44 years) and SLL/CLL (55-74 years).

Continues (Appendix 2)

Appendix 2. Continued...

Skin melanoma[a]

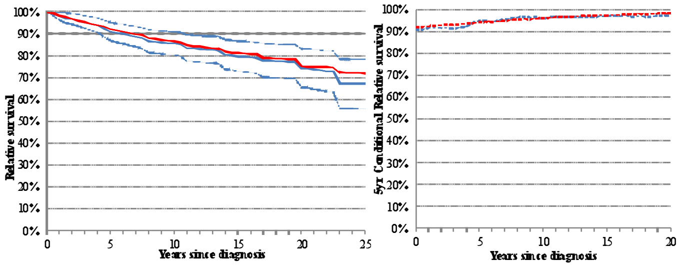

Cases contributing to estimates

2506 2035 1281 729 299 59

Mesothelioma

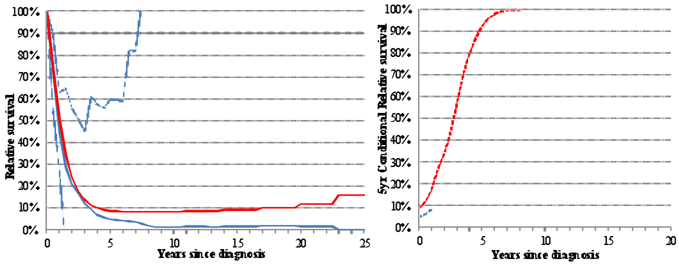

Cases contributing to estimates

294 19 9 5 3 2

Connective tissue

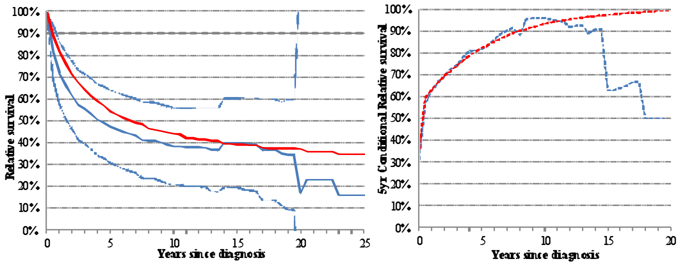

Cases contributing to estimates

413 170 92 56 20 6

Breast

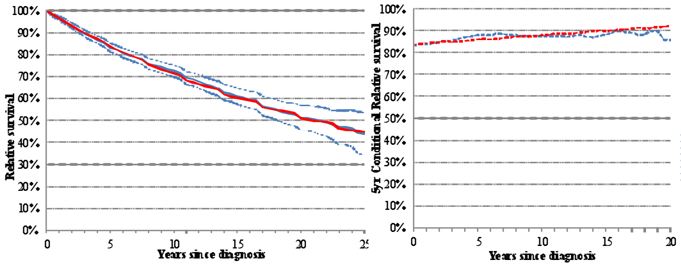

Cases contributing to estimates

23871 17100 9712 4262 1056 103

Cervix uteri[a]

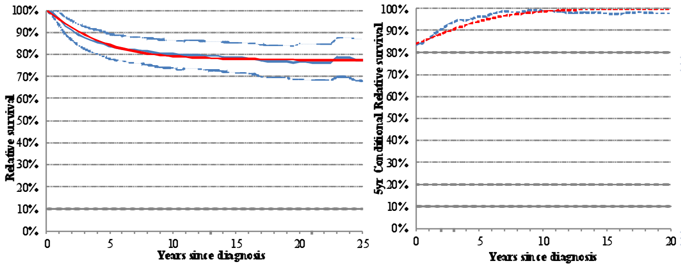

Cases contributing to estimates

1593 1196 858 577 272 89

[a] 65-74 years but Skin melanoma, Cervix uteri, Thyroid and Hodgkin lymphoma (15-44 years) and SLL/CLL (55-74 years).

Corpus uteri

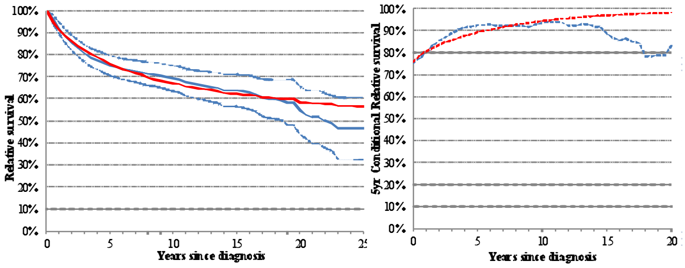

Cases contributing to estimates

4883 3100 1842 935 246 33

Ovary

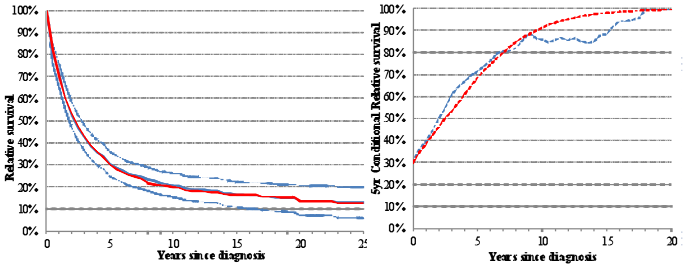

Cases contributing to estimates

2897 824 362 160 45 10

Kidney

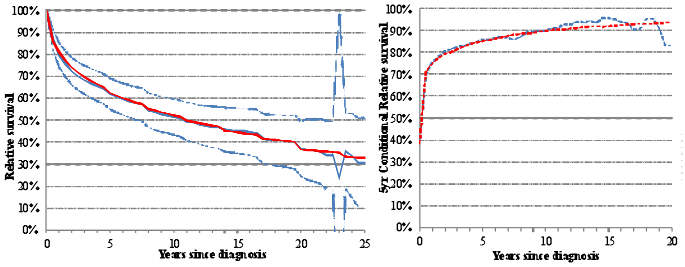

Cases contributing to estimates

2526 1415 779 355 79 7

Bladder

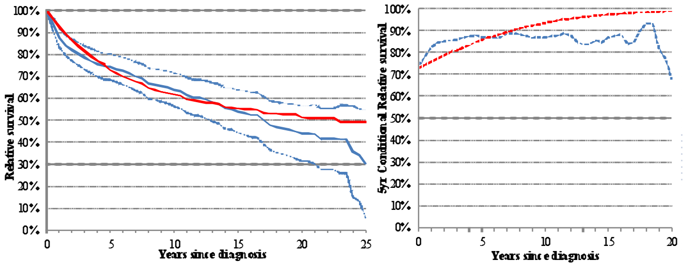

Cases contributing to estimates

2962 1936 1126 512 132 11

Brain

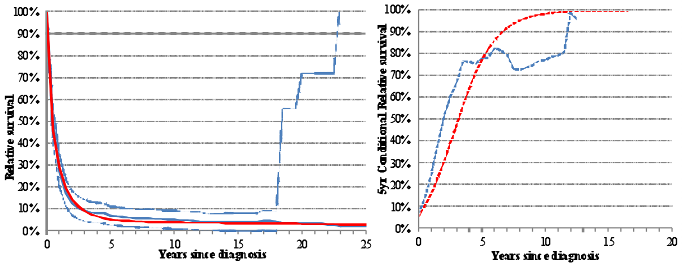

Cases contributing to estimates

1408 89 40 18 8 2

Continues (Appendix 2)

Appendix 2. Continued...

Thyroid[a]

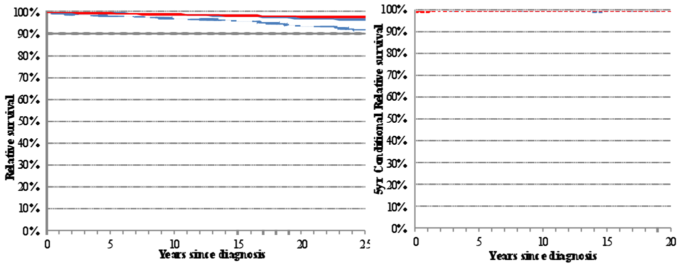

Cases contributing to estimates

3539 2883 1803 971 399 117

Non-Hodgkin lymphoma, DLBC

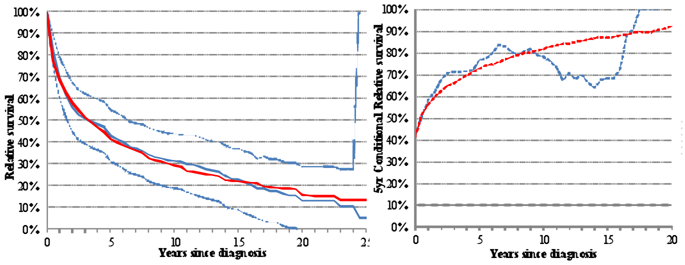

Cases contributing to estimates

956 378 166 67 11 3

Hodgkin lymphoma[a]

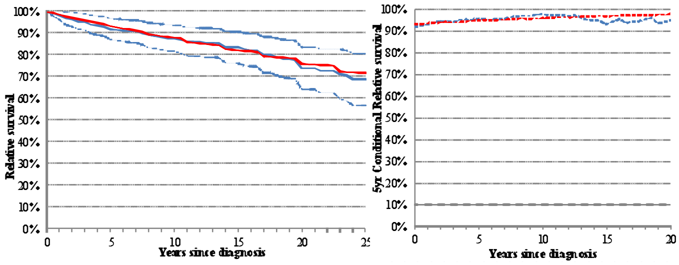

Cases contributing to estimates

1253 1049 750 469 210 56

Non-Hodgkin lymphoma, Follicular

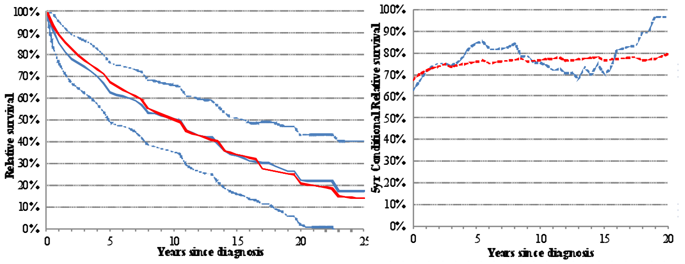

Cases contributing to estimates

566 314 163 62 15 3

Non-Hodgkin lymphoma

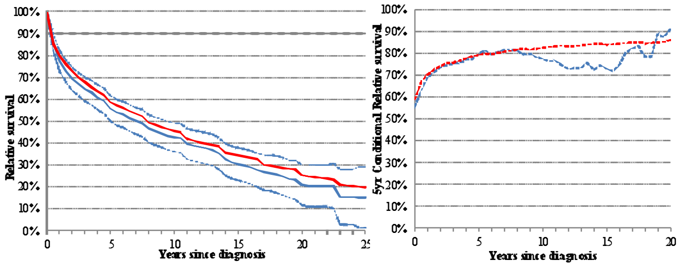

Cases contributing to estimates

3563 1803 889 356 81 6

Acute myeloid leukemia

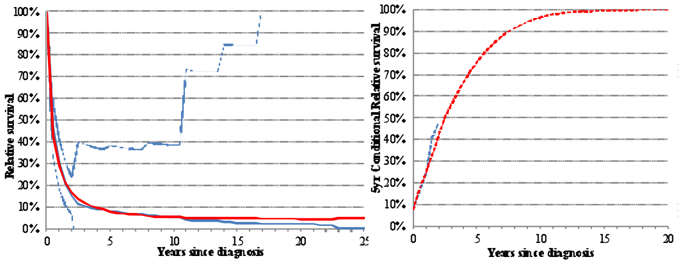

Cases contributing to estimates

622 53 21 9 5 2

Non-Hodgkin lymphoma, SLL/CLL[a]

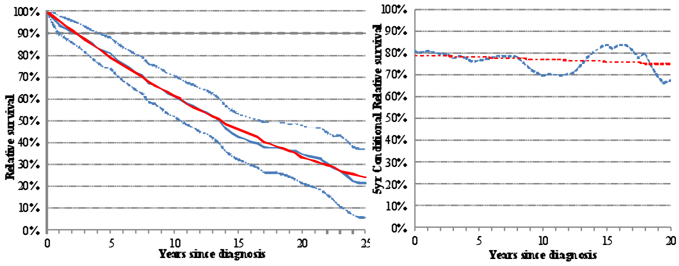

Cases contributing to estimates

1559 1126 627 274 73 10

Multiple myeloma

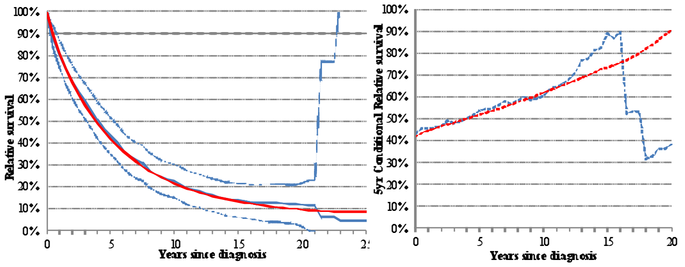

Cases contributing to estimates

1656 634 218 70 16 5

[a] 65-74 years but Skin melanoma, Cervix uteri, Thyroid and Hodgkin lymphoma (15-44 years) and SLL/CLL (55-74 years).

Appendix 3. Median life expectancy of fatal tumors (years)[a] at diagnosis by cancer type, sex, age, and year of diagnosis in Italy

Cancer type	MEN										WOMEN									
Age (years)	15-74		15-44		45-54		55-64		65-74		15-74		15-44		45-54		55-64		65-74	
Year of diagnosis	1990	2000	1990	2000	1990	2000	1990	2000	1990	2000	1990	2000	1990	2000	1990	2000	1990	2000	1990	2000
Oral cavity and pharynx	1.9	2.2									3.8	4.2								
Esophagus	0.6	0.7	0.8	1.0	0.8	0.9	0.6	0.8	0.5	0.7	0.7	0.8	0.8	0.8	0.9	0.9	0.7	0.9	0.6	0.8
Stomach	0.6	0.7	1.3	1.2	0.9	1.0	0.7	0.8	0.5	0.6	0.7	0.8	0.9	0.9	1.0	1.1	0.8	0.9	0.7	0.7
Colon	1.4	1.6	1.7	1.8	1.6	1.7	1.6	1.8	1.3	1.4	1.5	1.6	1.7	1.8	1.7	1.9	1.5	1.7	1.3	1.5
Rectum	2.0	2.2	2.4	2.5	2.0	2.1	2.2	2.4	1.8	2.0	1.9	2.1	2.2	2.2	2.3	2.5	2.1	2.2	1.7	1.9
Liver	0.4	0.7	0.6	0.7	0.4	0.8	0.4	0.8	0.4	0.7	0.5	0.8	0.8	0.9	0.4	0.9	0.6	0.8	0.4	0.8
Gallbladder	0.4	0.6	0.9	0.8	0.6	0.8	0.5	0.7	0.4	0.5	0.4	0.4	0.9	0.9	0.5	0.7	0.4	0.5	0.3	0.4
Pancreas	0.3	0.4	0.4	0.6	0.3	0.4	0.3	0.4	0.3	0.3	0.4	0.5	0.6	0.7	0.6	0.7	0.4	0.6	0.3	0.4
Larynx	10.9	10.8									10.1	13.6								
Lung	0.5	0.6	0.7	0.7	0.6	0.7	0.6	0.7	0.5	0.6	0.5	0.7	0.8	0.9	0.7	0.9	0.6	0.8	0.4	0.6
Skin melanoma	2.5	2.8	3.1	3.2	2.9	3.3	2.5	2.7	2.1	2.5	4.3	4.6	5.3	5.6	4.1	4.3	5.0	5.2	3.9	4.3
Mesothelioma	0.9	1.0	1.2	1.2	1.1	1.3	1.0	1.1	0.8	0.9	1.0	1.1	0.9	3.5	1.1	1.2	1.1	1.2	0.9	1.0
Connective tissue	2.2	2.4									2.0	2.1								
Breast											6.6	7.2	10.5	13.0	8.3	9.6	8.5	10.3	8.1	9.4
Cervix uteri											2.4	2.5	2.7	2.7	2.0	2.1	3.3	3.5	2.7	2.8
Corpus uteri											3.9	4.0	6.3	6.2	2.8	2.8	6.5	6.8	3.5	3.6
Ovary											1.8	2.0	2.6	2.7	2.6	2.8	2.2	2.4	1.4	1.6
Prostate	5.2	7.4																		
Testicular	9.8	10.6	5.8	6.1	6.1	6.0	>15	>15	5.6	5.3										
Kidney	8.5	>15	>15	>15	8.5	12.0	10.8	>15	6.5	14.9	3.2	4.2	2.1	2.2	3.3	4.1	4.6	6.3	3.0	3.9
Bladder	>15	>15	6.9	7.1	6.2	6.5	5.5	6.0	4.3	4.7	>15	>15	2.4	2.5	2.3	2.3	4.9	5.0	3.3	3.5
Brain	0.5	0.6	2.2	2.5	1.0	1.1	0.7	0.8	0.4	0.5	0.4	0.6	2.7	2.9	1.0	1.3	0.6	0.8	0.4	0.4
Thyroid	7.0	>15	3.6	3.7	8.2	9.1	2.5	3.1	2.2	3.1	>15	>15	4.6	4.6	5.3	5.6	3.8	4.1	2.0	2.5
Hodgkin lymphoma	6.1	12.9	6.3	6.6	6.3	7.3	5.5	7.5	2.9	3.3	8.7	>15	6.9	7.5	10.3	12.1	7.5	9.4	4.3	6.0
Non-Hodgkin lymphoma	3.1	5.0									5.4	9.7								
SLL/CLL[b]	7.8	9.1			8.5	10.9			3.6	3.6	12.1	>15			13.4	>15			5.1	4.9
NHL, diffuse large B	0.9	1.2	0.7	0.8	1.6	1.8	1.3	2.0	0.9	1.2	1.8	2.1	1.1	1.2	6.7	7.6	2.8	3.5	1.6	2.1
NHL, follicular	6.6	11.8									6.6	12.9								
Multiple myeloma	3.0	4.2	2.6	3.8	4.0	5.7	3.4	5.0	2.8	3.5	3.7	4.7	3.9	5.9	3.8	5.7	3.9	4.8	3.4	3.4
Acute myeloid leukaemia	0.3	0.5	0.8	0.9	0.5	0.6	0.3	0.5	0.3	0.4	0.3	0.5	0.8	0.9	0.6	0.9	0.5	0.8	0.3	0.4
**All types**	1.0	1.4	1.5	1.6	1.2	1.4	1.1	1.5	1.0	1.4	2.3	2.7	3.7	4.1	3.0	3.3	2.6	3.1	1.7	2.0

[a]Median life expectancy of fatal tumors at diagnosis was calculated in years as the median (50th percentile) RS estimated through the best fitting model-based distributions.
[b]Estimates for patients aged 15-54 and 55-74 years

Appendix 4. Estimated cure fraction (%) by cancer type, sex, age, and calendar year of diagnosis in Italy.

Cancer type	MEN								WOMEN							
**Age (years)**	**15-44**		**45-54**		**55-64**		**65-74**		**15-44**		**45-54**		**55-64**		**65-74**	
**Year of diagnosis**	**1990**	**2000**	**1990**	**2000**	**1990**	**2000**	**1990**	**2000**	**1990**	**2000**	**1990**	**2000**	**1990**	**2000**	**1990**	**2000**
Oral cavity and pharynx	25	32	17	24	12	18	8	14	37	43	28	33	22	27	16	21
Esophagus	6	11	4	9	3	6	3	6	51	39	10	11	5	10	7	11
Stomach	41	34	30	33	20	28	12	17	35	36	31	34	28	33	19	23
Colon	56	61	48	58	44	56	42	52	53	63	47	59	51	62	44	53
Rectum	50	54	42	54	37	49	35	45	54	55	44	55	43	58	37	50
Liver	11	16	1	6	1	4	0	2	14	20	1	9	3	6	0	2
Gallbladder	41	30	11	17	7	13	5	8	28	36	10	17	6	12	5	7
Pancreas	5	11	2	4	2	4	2	3	11	19	5	10	2	6	2	4
Larynx	28	27	22	21	18	18	15	14	29	29	22	22	18	17	14	14
Lung	14	21	8	11	6	9	4	6	18	30	11	17	8	14	4	9
Skin melanoma	70	84	58	76	56	73	41	67	76	88	77	85	72	79	57	74
Mesothelioma	23	23	4	8	1	2	1	2	3	25	10	13	7	8	3	6
Connective tissue	59	63	49	53	41	46	33	39	58	64	49	56	43	50	36	44
Breast									57	71	62	77	50	68	40	54
Cervix uteri									78	80	64	70	49	55	36	39
Corpus uteri									77	77	85	86	64	69	57	62
Ovary									62	62	35	44	24	31	15	22
Prostate	41	80	37	78	34	76	31	75								
Testicular	91	96	95	93	70	86	70	52								
Kidney	63	74	41	54	29	45	22	35	68	83	48	63	37	51	32	42
Bladder	83	89	67	74	51	62	42	53	88	94	80	84	68	72	51	61
Brain	23	30	8	11	4	8	2	3	26	30	9	17	5	10	3	4
Thyroid	91	97	68	84	42	79	25	65	98	99	88	98	77	93	47	86
Hodgkin lymphoma	75	83	51	71	16	38	22	35	74	88	58	80	27	48	13	34
Non-Hodgkin lymphoma	35	49	25	40	20	34	15	28	46	59	31	46	22	36	14	27
SLL/CLL[a]							2	5							6	10
NHL, diffuse large B	45	61	41	52	19	31	17	25	52	62	41	46	24	34	13	21
NHL, follicular	43	61	23	43	13	30	5	18	54	73	33	57	20	44	9	30
Acute myeloid leukaemia	30	51	22	38	4	12	1	3	29	47	10	28	5	14	3	6
Multiple myeloma	21	63	8	20	3	10	3	5	25	68	13	40	5	10	7	7
**All types**	**54**	**67**	**32**	**45**	**23**	**38**	**20**	**34**	**59**	**71**	**53**	**65**	**40**	**52**	**30**	**39**

[a]Estimates for patients aged 55-74 years.
